# Pulmonary Inflammatory Myofibroblastic Tumor: A Case Report

**DOI:** 10.1055/a-2430-0053

**Published:** 2024-11-04

**Authors:** Lotte Bruyninckx, Paul De Leyn, Dirk Van Raemdonck, Yanina Jansen, Katrien Coppens, Francois Vermeulen, Birgit Weynand, Christopher Gieraerts, Herbert Decaluwé

**Affiliations:** 1 Department of Thoracic Surgery, University Hospitals Leuven, Leuven, Belgium; 2 Department of Paediatrics, Imelda Hospital, Bonheiden, Belgium; 3 Department of Paediatrics, University Hospitals Leuven, Leuven, Vlaams-Brabant, Belgium; 4 Department of Pathology, University Hospitals Leuven, Leuven, Vlaams-Brabant, Belgium; 5 Department of Radiology, Imelda Hospital, Bonheiden, Belgium; 6 Department of Thoracovascular Surgery, Ziekenhuis Oost-Limburg, Genk, Limburg, Belgium

**Keywords:** inflammatory myofibroblastic tumor, histopathology, anaplastic lymphoma kinase, pediatric surgery

## Abstract

An inflammatory myofibroblastic tumor (IMT) is a rare mesenchymal tumor that occurs predominantly in children and young adults. Etiology remains unclear. But based on the frequent detection of chromosomic alterations, especially near the anaplastic lymphoma kinase (ALK) gene, IMT is now considered to be a true neoplasm. In addition, the possible aggressive behavior, and the ability to metastasize suggest at least an intermediate malignant potential. Surgery remains the treatment of choice, but the use of chemotherapy, nonsteroidal anti-inflammatory drugs, immunotherapy, and targeted therapy are reported. We describe a case of a pulmonary IMT in a 6-year-old boy with an incidental finding of a lesion in the right upper lobe. A video-assisted thoracoscopic right upper lobectomy with lymph node resection was performed. Microscopic examination confirmed the diagnosis of IMT with the nodule showing spindle cells in a background of plasma cells. ALK immunohistochemical expression was negative.

## Introduction

An inflammatory myofibroblastic tumor (IMT) is a soft tissue neoplasm that predominates in children and young adults. 1 2 3 In total, 50% of all documented benign primary pulmonary neoplasms in a pediatric population are IMTs. 1 Nevertheless, pulmonary IMT still remains a unique presentation, given the rarity of primary pulmonary tumors in this population in general.

IMT was first described in 1939 as a benign lung tumor with contradictory histopathology. 1 Given its fibro-inflammatory aspect and the believe it originated from a post-inflammatory repair process, it was later classified under the umbrella term “inflammatory pseudotumor (IPT).” 3 Afterwards, the origin of IMT remained debatable for decades. At the end of the 1990s, the detection of chromosomal abnormalities made it clear that IMT must be seen as a true neoplasm. 4 5 6 First, it was classified as a benign neoplasm, but nowadays the World Health Organization (WHO) describes IMT as a lesion of intermediate malignant potential. 7

We present a case of a young boy with pulmonary IMT.

## Case Report

A 6-year-old boy with hyperreactive airways and a history of prolonged post-viral coughing presented with recurrent respiratory infections for over a month. Since the age of 2, he received a fluticasone/propionate inhaler as maintenance therapy.

**Fig. 1:**
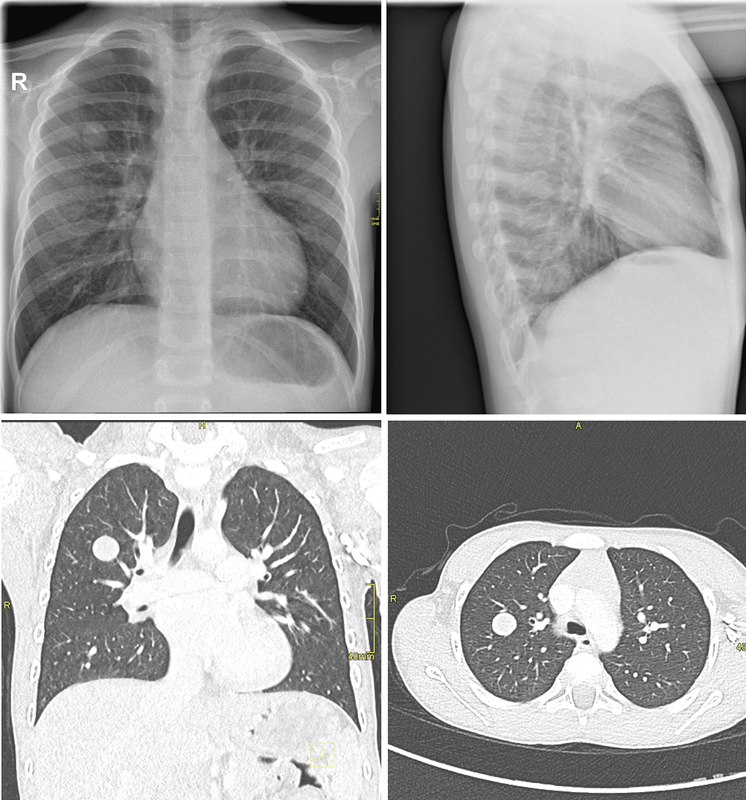
Chest X-ray and computed tomography (CT) scan showing a lesion in the right upper lobe.

In the weeks prior to diagnosis, he suffered from two respiratory tract infections with high fever and myalgia. Each was followed by a prolonged period of severe coughing, with the child remaining pale and weakened afterwards. A few cervical lymph nodes were noticed. Lung auscultation was normal. The laboratory tests were compatible with a viral infection: leukopenia with lymphocytosis and normal C-reactive protein. A chest radiograph showed an image of bronchitis and a lesion of 18 mm in the right upper lobe ( Fig. 1 ). The lesion was absent on a chest radiograph 3 years earlier.

A computed tomography (CT) scan was performed, which showed a well-defined solitary nodule of 16 mm in the right upper lobe ( Fig. 1 ). Radiological differential diagnosis suggested a hamartoma, carcinoid tumor or metastasis. An abdominal ultrasound, serum tumor markers, and a catecholamine dosage on 24-hour urine were normal with the exception of a slightly elevated neuron-specific enolase (21 µg/L).

A whole-body CT scan and a positron emission tomography scan were conducted, with the lesion showing a selective hypermetabolism, and therefore suggesting a malignant entity. Meanwhile, the clinical image resolved, and the patient could resume his normal activities.

One month after first presentation, a video-assisted thoracoscopic (VATS) right upper lobectomy with lymph node resection was performed. The patient weighted 23.7 kg at the time of surgery. The procedure was achieved by an uniportal incision above the sixth rib. The surgeon used 3 and 5 mm material. Segmental arteries were either stapled or coagulated. Upper lobe vein and bronchus were stapled. The total operative time was 2 hours and 33 minutes. There were no postoperative complications and 4 days after surgery, the patient was discharged.

**Fig. 2:**
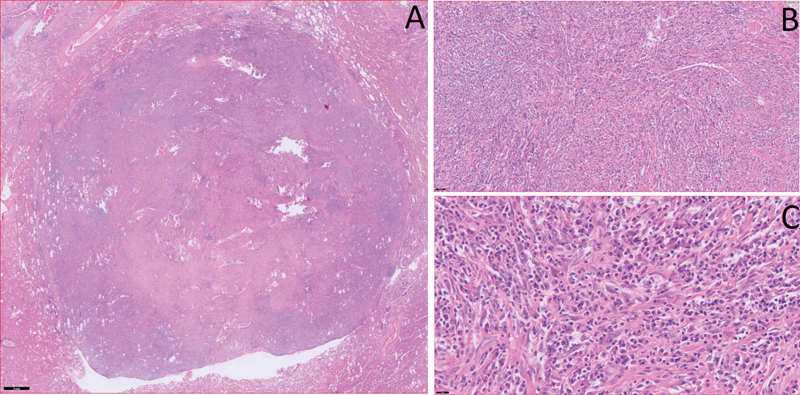
Histopathological examination of the lesion in different magnifications: ( **A** ) nonencapsulated, well-circumscribed tumor nodule surrounded by normal lung parenchyma (HE staining, Bar = 1 mm). ( **B** ) On higher magnification, the tumor is made of fibrous fascicules mixed with chronic inflammatory cells and a few vessels (HE staining, bar = 50 micron). ( **C** ) On highest magnification, the tumor is made of fibrous fascicles mixed with numerous plasma cells and a few eosinophils. No atypia, no significant mitotic activity (HE staining, bar = 20 microns). Inclusion of a few normal alveolar structures are not shown.

On histopathological examination, the lesion was 17 mm in size, tan and firm with a smooth surface. Tumor-free section margins were at least 5 mm. All resected lymph nodes were found to be negative. Microscopic examination of the nodule showed spindle cells in a background of plasma cells ( Fig. 2 ). Foamy histiocytes, lymphocytes, and eosinophils were sporadically seen. No nuclear atypia or elevated mitotic activity was observed. Anaplastic lymphoma kinase (ALK) immunohistochemical expression was negative. KI67 staining was not performed. Based on these data, the diagnosis of plasma cell granuloma IMT was made.

The follow-up period lasted one and a half years, during which no local or distant recurrence was observed.

## Discussion and Conclusion

We describe an incidental finding of IMT in the right upper lobe in a 6-year-old boy.

Although IMT occurs at any age, it predominately occurs in children and adolescents. 1 2 3 8 Lesions are typically 10 to 60 mm in size, round, well-demarcated, and slowly growing. 2 They are mostly found in the lungs but can develop in any organ, including the mediastinum, urinary bladder, ovary, liver, kidney, gastrointestinal tract, and soft tissues. 2 8 9 When in the lung, parenchymal IMT generally outweighs the prevalence of endobronchial lesions. 2 4

Symptoms depend strongly on the tumor's location. 2 Pulmonary IMT usually presents with fever and cough, often accompanied by respiratory distress, arthralgia, clubbing, night sweats, vomiting, and hemoptysis. 10 Central IMTs tend to be recognized earlier. 11 Frequently IMT is a coincidental finding, as many patients remain asymptomatic. 8

While spontaneous regression has been reported, the therapy of choice remains surgical excision. 3 9 12 In this case, a VATS lobectomy was combined with systematic lymph node dissection as mediastinal metastases have been described. 13 A complete resection is considered curative with no adjuvant treatment necessary, as local recurrence after radical excision is a rare event. 2 9 10 14 Chemotherapy is generally ineffective but could be indicated when IMT is complicated with local invasion or recurrence. 6 8 14 ALK inhibitors can provide durable responses in unresectable or metastatic ALK-expressing IMT, although relapses due to secondary ALK mutations have been reported. 14 15 16 Treatments with immunotherapy, nonsteroidal anti-inflammatory drugs, and steroids are reported with various results. 10 16 17

Histologically, IMT consists of three different presentations (xanthogranuloma, plasma cell granuloma, and sclerosing pseudotumor type), but these patterns are of no clinical importance and can even coexist in the same lesion. 18 Because of this fibro-inflammatory aspect, IMT was long considered an IPT. Presently, it is believed that IMT is a true neoplasm. 4 5 6 The etiology remains unclear. Griffin et al discovered in 1999 recurrent abnormalities on chromosome 2 at 2p23 with alterations near or within the ALK gene. 4 5 Many kinds of chimeric ALK genes have been reported so far. 19 These translocations result in an overexpression of tyrosine kinase receptor ALK. Anti-ALK antibodies, a specific marker for abnormal ALK gene expression by immunohistochemistry, are detected in 50% of all IMTs. 11 14

Expression of ALK is higher in younger patients and in extrapulmonary IMT. 8 14 The presented case lacked ALK expression, aligning with Camela et al's study, which found that only 16% of reviewed pulmonary IMTs were ALK-positive. 10

ALK-positive tumors have a less than 5% risk of metastasis, as opposed to ALK-negative lesions, which are more prone to distant spread. 6 14 Despite ALK-positive lesions being more locally aggressive and have a tendency to local recurrence, ALK expression is considered a favorable prognostic indicator due to the rarity of local recurrence after radical excision. 9 10 17

These chromosomal alterations, in combination with a more locally aggressive behavior of ALK-positive IMT, have led to the belief that IMT is a true neoplasm. 5 14 Currently the WHO describes IMT as a lesion of intermediate malignant potential, because of the risk of local recurrence and distant metastasizing. 7 Long-term follow-up remains indicated, as cases of relapse are described several years after primary surgery. 2 There are no established guidelines for pulmonary pediatric IMT follow-up; therefore, we propose a protocol based on standard oncology protocols, using magnetic resonance imaging (MRI) to minimize radiation exposure: a chest X-ray at 3 and 6 months postresection, followed by yearly MRI starting 1 year after surgery.

Pulmonary IMT is a rare disease and could be missed given the nonspecific symptoms at presentation, especially in peripheral lesions. It should always be considered in the differential diagnosis of a primary pulmonary lesion in children and young adults. To confirm the diagnosis, histopathological examination is mandatory. Primary surgery is the treatment of choice with a good overall survival rate in case of complete resection. Nevertheless, prolonged follow-up of these patients remains necessary due to the intermediate malignant potential.

## Notes

Importance for a Pediatric Surgeon ALK inhibitors show promising results in ALK-expressing IMT. Nevertheless, primary surgery is the treatment of choice with a good overall survival rate in case of complete resection. But prolonged follow-up is mandatory, as cases of delayed metastasis are described.
